# Electrical detection of RNA cancer biomarkers at the single-molecule level

**DOI:** 10.1038/s41598-023-39450-6

**Published:** 2023-08-01

**Authors:** Keshani G. Gunasinghe Pattiya Arachchillage, Subrata Chandra, Ajoke Williams, Patrick Piscitelli, Jennifer Pham, Aderlyn Castillo, Lily Florence, Srijith Rangan, Juan M. Artes Vivancos

**Affiliations:** Department of Chemistry, University of Massachusetts Lowell, Lowell, MA 01854 USA

**Keywords:** Bioenergetics, Nanoscale biophysics, Single-molecule biophysics, Scanning probe microscopy, Sensors, Biophysical chemistry, Electron transfer, Electrical and electronic engineering, DNA and RNA, Electronic properties and materials

## Abstract

Cancer is a significant healthcare issue, and early screening methods based on biomarker analysis in liquid biopsies are promising avenues to reduce mortality rates. Electrical detection of nucleic acids at the single molecule level could enable these applications. We examine the electrical detection of RNA cancer biomarkers (KRAS mutants G12C and G12V) as a single-molecule proof-of-concept electrical biosensor for cancer screening applications. We show that the electrical conductance is highly sensitive to the sequence, allowing discrimination of the mutants from a wild-type KRAS sequence differing in just one base. In addition to this high specificity, our results also show that these biosensors are sensitive down to an individual molecule with a high signal-to-noise ratio. These results pave the way for future miniaturized single-molecule electrical biosensors that could be groundbreaking for cancer screening and other applications.

## Introduction

**Figure 1:**
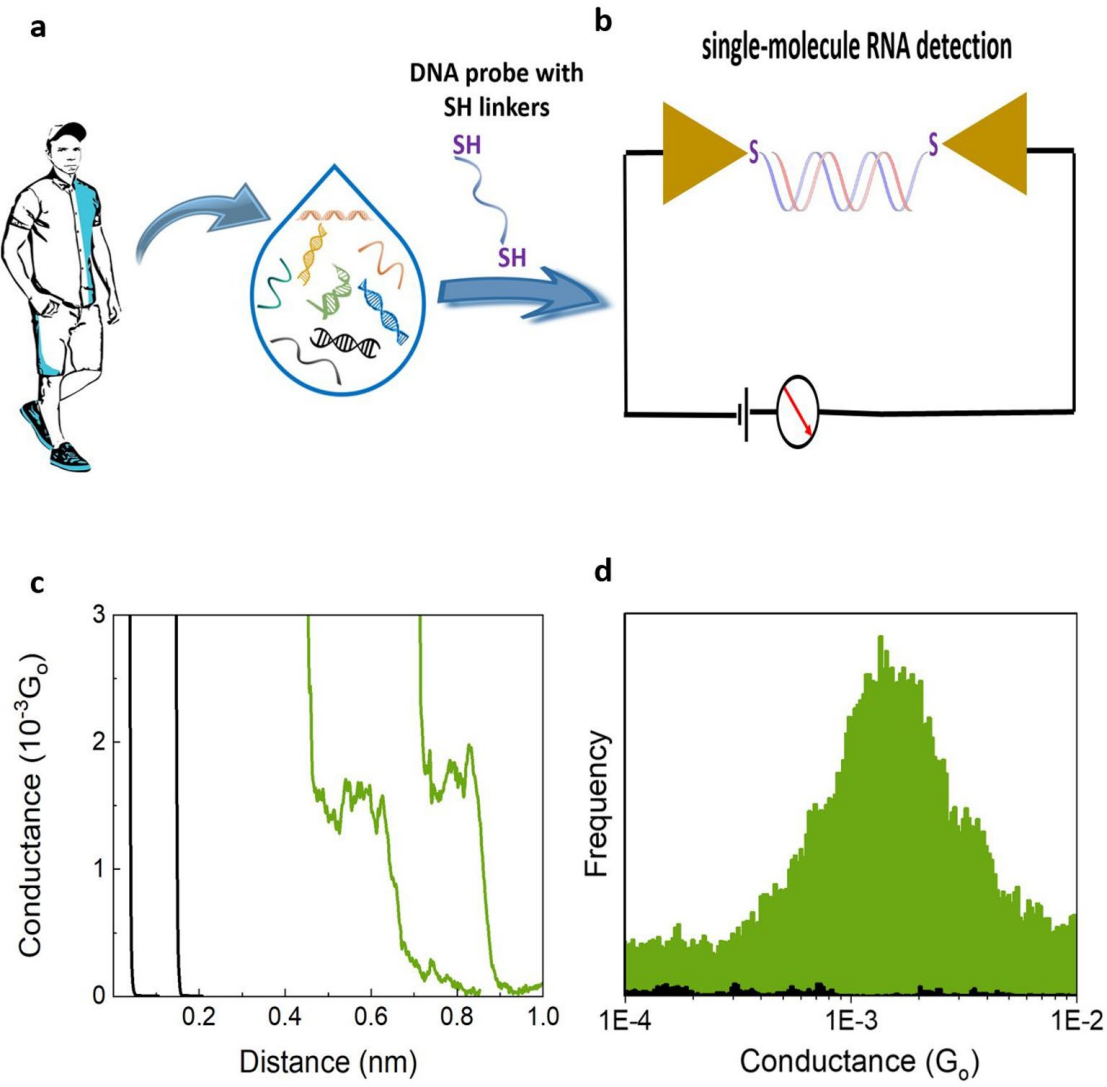
Detection of cancer biomarkers using the STMBJ single-molecule conductance approach^18^. (**a**) Liquid biopsy samples contain circulating nucleic acids that can be detected with a complementary DNA probe capable of binding to STM electrodes. (**b**) STMBJ detection of the hybridized biomarker, resulting in a step in the conductance–distance signal. (**c**) Example of collected conductance–distance curves. Control phosphate buffer solution (black), green: example raw data curves collected from the G12C sample. (**d**) Histogram built from curves with steps (black: Phosphate buffer blank, green: G12C RNA sample), 118 from a total 5399 curves acquired in the experiment.

According to the World Health Organization, cancer is one of the leading causes of death, which accounted for approximately 10 million deaths in 2020 and is still on the rise^1–3^. Early cancer detection through liquid biopsies and noninvasive analysis of cancer biomarkers in body fluids could help reduce these mortality rates^4^. Liquid biopsy approaches target cancer-specific biomarkers in samples such as blood or saliva, including tumor cells or circulating tumor nucleic acids (circulating tumor DNA, ctDNA, and RNA, ctRNA)^5^. Detecting ctNA in liquid biopsy samples is challenging due to the low concentration of ctNA and the low frequency of mutations compared to wild-type sequences^5–9^. Advances in nanoscience and nanotechnology could help address these challenges^5–8^. In particular, the Scanning Tunneling Microscopic (STM)-assisted break junctions method (STMBJ)^10^ has recently allowed the first single-molecule detection and identification of RNA from E. coli strains^11^, between several other single-molecule electrical studies on oligonucleotides^12–17^. This indicates that the same approach could be used for cancer biomarker RNA sequences from human origin^18^, more specifically, ctNAs could be targeted by their corresponding mutations that are biomarkers associated with cancer risk. These biomarkers could be detected by designing DNA probes complementary to the target sequence and modified with chemical groups that allow binding to electrodes (i.e., thiol binding groups; see Fig. 1). From all the NAs present in a sample, the DNA probe could target ctRNA sequences more efficiently because during the interphase of the cell cycle, RNA transcription naturally amplifies those RNA targets^19^ avoiding the amplification or cell culturing steps^11,20^. This could be a suitable method for liquid biopsy, as it is capable of detecting small amounts of RNA biomarkers in solution by electrical means, providing sequence-specific electrical fingerprints, being sensitive to single-point mutations^11,12^, and amenable for future miniaturization and automation. Herein, we show proof-of-concept STMBJ measurements for such a biosensor. These constitute the first single-molecule conductance results for sequences from human origin.

Figure 1 shows the potential strategy for this detection method; a liquid biopsy sample containing multiple ctNA (Fig. 1a) can be targeted with specific dithiol-modified DNA probes for single-molecule electrical detection of the RNA cancer biomarker (Fig. 1b). When the DNA probe hybridizes with the target RNA, resulting in a DNA:RNA hybrid that can bind to the STM electrodes^11,14,15^, the biomolecular electronics circuit is “closed” and electrical fingerprints can be measured (molecular conductance steps in the STMBJ conductance–distance curves in Fig. 1c)^11,14,15^. Repeating this STMBJ experiment thousands of times, we can accumulate enough single-molecule electrical fingerprints to perform a statistical analysis, resulting in a conductance histogram showing the most likely conductance value for this particular DNA:RNA hybrid (Fig. 1d).

In this study, we performed STMBJ experiments that demonstrate the electrical detection of RNA cancer biomarkers. We measured the conductance of the G12C and G12V KRAS mutations, and in both cases, we can differentiate the mutation from the wt KRAS sequence with a four-time signal difference. Furthermore, we can simultaneously measure the mutant cancer biomarkers in the presence of the wild-type form in solution. Titration experiments also show a low limit of detection for this proof-of-concept electrical biosensor, effectively at the single-molecule level with a signal-to-noise ratio (SNR) around four. Our results pave the way for rapid and early electrical screening of cancers with high sensitivity and specificity.

## Results and discussion

### Design of biomolecular electronics DNA probes through bioinformatics

Recent literature includes reports on an unprecedented number of genomic studies on cancer biomarkers^21–23^. In particular, Pancancer Analysis of Whole Genomes (PCAWG), the Consortium of the International Cancer Genome Consortium (ICGC), and Cancer Genome Atlas (TCGA) analyzed 2658 whole cancer genomes from 38 tumor types^21^, and this creates the opportunity to identify potential mutant sequences for RNA cancer biomarkers to be targeted in liquid biopsy samples. The viral oncogene homolog of Kirsten rat sarcoma (KRAS) is a well-known oncogene with a high mutation rate in several cancers^24^. KRAS mutations are frequently found in pancreatic ductal adenocarcinoma, non-small cell lung cancer, colorectal cancer and skin melanoma^22,24^. KRAS mutations are usually single base mutations, and 98% of them occur at codon 12 (G12)^24,25^. Among G12, the G12C substitution is present in 46% of all lung adenocarcinomas^25^, while G12V is present in many colorectal and pancreatic cancers^26^. We decided to focus on these two KRAS mutations for our proof of concept study, as they cover several types of cancer and show promise for future cancer screening approaches^22^.

**Figure 2:**
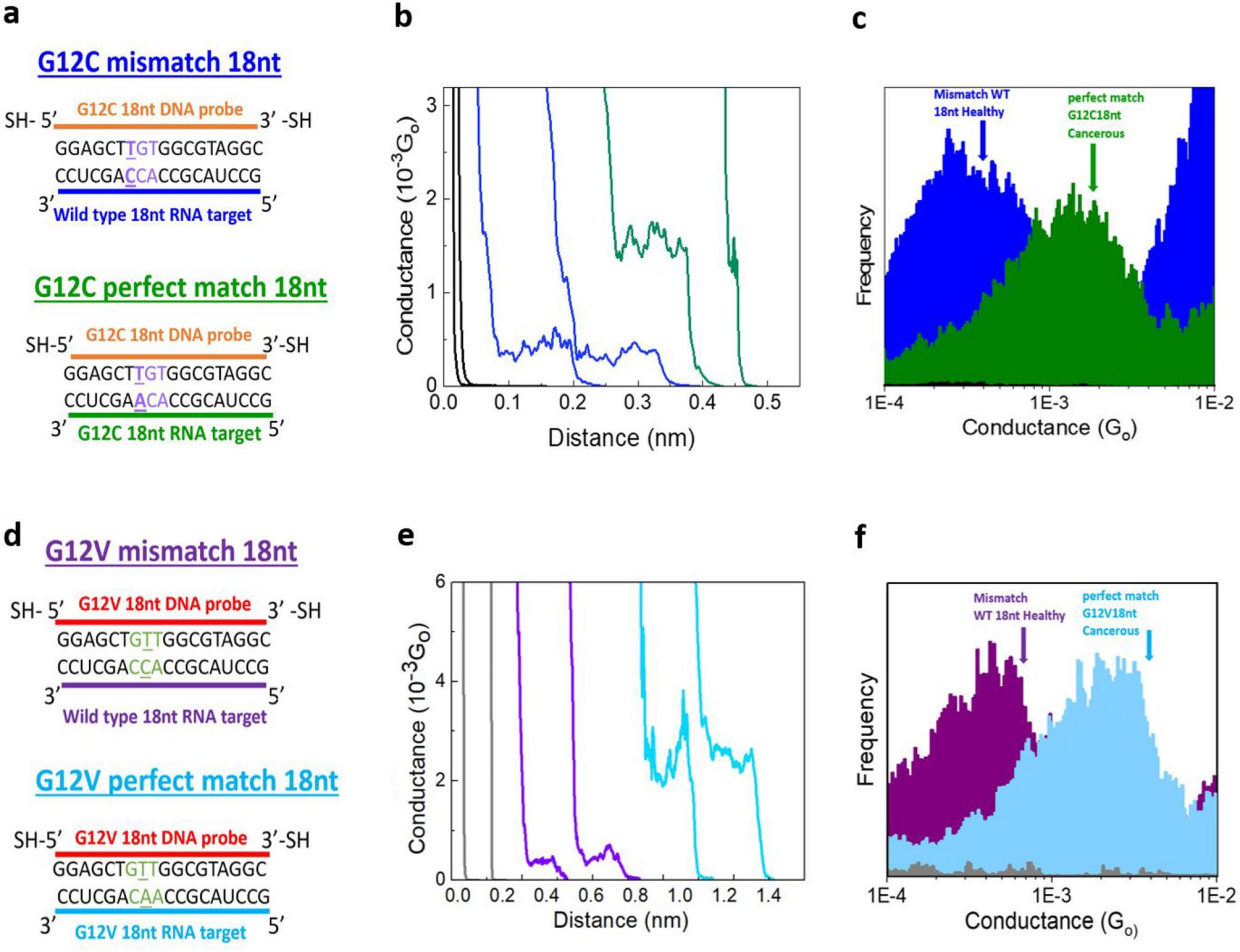
Electrical detection of KRAS mutations. (**a**) Sequences for G12C 18nt mismatch and perfect match. (**b**) Example conductance versus distance curves (Black: blank, Blue: mismatch, Green: perfect match). (**c**) Histograms for G12C mismatch (379 from a total 5203 curves acquired) and perfect match (212 from a total 13,958 curves acquired) overlapped with phosphate buffer blank (Blue: G12C 18nt mismatch, Green: G12C 18nt perfect match, Black: phosphate buffer blank). (**d**) Sequences for G12V 18nt mismatch (healthy) and perfect match (cancerous). (**e**) Raw conductance versus distance curves (Black: blank, Purple: mismatch, Light blue Blue: perfect match). (**f**) Histograms for G12V mismatch (75 from a total 5024 curves acquired) and perfect match (116 from a total 10,159 curves acquired) overlapped with phosphate buffer blank (Purple: G12V 18nt mismatch, Light blue: G12V 18nt perfect match, Gray: phosphate buffer blank).

To select the appropriate sequences for the study, we focus on KRAS Exon 2, comparing the wild-type sequence with the G12C and G12V mutations. For single-molecule electrical conductance applications, it is important to find a balance in sequence length; shorter sequences will result in higher conductance^14,27–29^ (higher signal-to-noise ratio), but a minimum length is required to ensure specificity for the KRAS (see supplementary Fig. S1). We used the NCBI blast tool to align candidate sequences ranging 20–10 bases (see supplementary Figs. S1 and S2), and found that the optimum short sequences that are still specific are 18 bases long^30^. These sequences were analyzed using other bioinformatic tools; % GC content, melting temperature, and potential secondary structures were obtained using the IDT OligoAnalyzer tool^31^ (results in supplementary Table S1). We selected sequences that have high melting temperatures and do not present significant secondary structures at room temperature. Fig. 2a and d show the RNA biomarker sequences with the resulting thiol-modified complementary DNA probes.

### Single-molecule cancer biomarker electrical detection

We use the STMBJ method^10^ to measure charge transport conductance values in individual KRAS G12C and G12V RNA molecules by forming single-molecule junctions in a nanogap formed between the STM electrodes. Briefly, by bringing an atomically sharp Au tip into and out of contact with an Au substrate electrode while the conductance is recorded, we can accumulate thousands of conductance–distance curves. When an individual biomolecule binds to both electrodes, a step is recorded that interrupts the otherwise exponential conductance–distance trace. We used the DNA probes discussed above, modified with thiol binding groups at the 3’ and 5’ ends, so they can bind to the electrodes and hybridize with the complementary target RNA sequence, closing the biomolecular electronics circuit and resulting in a particular conductance step. Figure 1c shows that “steps” are not observed when no molecules are bound between the two electrodes (see control experiment in phosphate buffer in the absence of RNA, black and gray low-intensity histograms in Figs. 1 and 2), while steps are observed when a single molecule is bound between the electrodes. By collecting thousands of conductance traces, a conductance histogram is generated using curves with steps to obtain the average conductance value for the particular sequence. Note that no steps are recorded when the DNA probe is measured in the absence of target RNA (see supplementary Fig. S3). Previous research shows that molecular conductance is highly sensitive to small changes in nucleotide sequence, even for a single base alteration^11,12^, and this indicates that this biosensing strategy should allow the differentiation of KRAS mutants from the wt KRAS sequence.

Figure 2 shows the results of the measurements on two biomarkers; G12C and G12V. Figure 2b represents in green example conductance–distance curves examples for the G12C sequence, while in blue it shows the same data for an experiment on the wild-type sequence that may hybridize with the KRAS G12C DNA probe, resulting in a single-base mismatch. The steps for the perfect match G12C DNA:RNA measurements are higher than those for mismatched sequences. In particular, the histograms in Fig. 2c show that the average conductance of G12C is four times higher than that of the mismatched wild-type sequence, allowing the discrimination of individual cancer biomarker molecules (green histogram) from the regular wt KRAS sequence (blue histogram), which will probably be present in any sample of human origin^32–34^. Similarly, Fig. 2(d–f) shows the electrical detection of the KRAS G12V mutation. We followed the same strategy as above for the perfect match (G12V mutant) and mismatch (wt KRAS) scenarios. Figure 2d shows the sequences and Fig. 2e the raw data conductance curves examples. Finally, Fig. 2f shows the resulting conductance histogram where, similarly to the G12C case above, the ratio between the biomarker and the wt KRAS mismatch signal is around four. The statistics resulting from three independent STMBJ experiments for each case are presented in Table 1.

**Table 1:** Statistics of single-molecule conductance for G12V and G12C mutations: average and standard deviations for three different single-molecule conductance experiments, from at least 4000 acquired curves ($G_0=2e^2/h$; (h: Planck constant, e: electron charge).

Sequence DNA:RNA	G12V:wtKRAS	G12V:G12V	G12C:wtKRAS	G12C:G12C
Average conductance ($10^{-4}G_0$)	5.6 ± 1.2	23 ± 2.2	3.9 ± 0.32	17 ± 2.6

These are the first single-molecule measurements of an RNA sequence of human origin and the first exploration of the electrical detection of individual cancer biomarker molecules. From the biosensing perspective, this indicates that single-molecule conductance from an accurately designed DNA probe to capture a NA biomarker can discriminate a cancer biomarker from the equivalent wild-type “healthy” sequence that differs in only one base with a fourfold separation between the signals. To further challenge this single-molecule biosensing method, we verify the specificity of the sensor by trying to *in situ* detect both sequences simultaneously, since in typical liquid biopsy samples, both mutant and wt KRAS sequences will be present in solution^32–34^.

### Specificity: simultaneous detection of KRAS mutations and single-base wild-type mismatches

**Figure 3:**
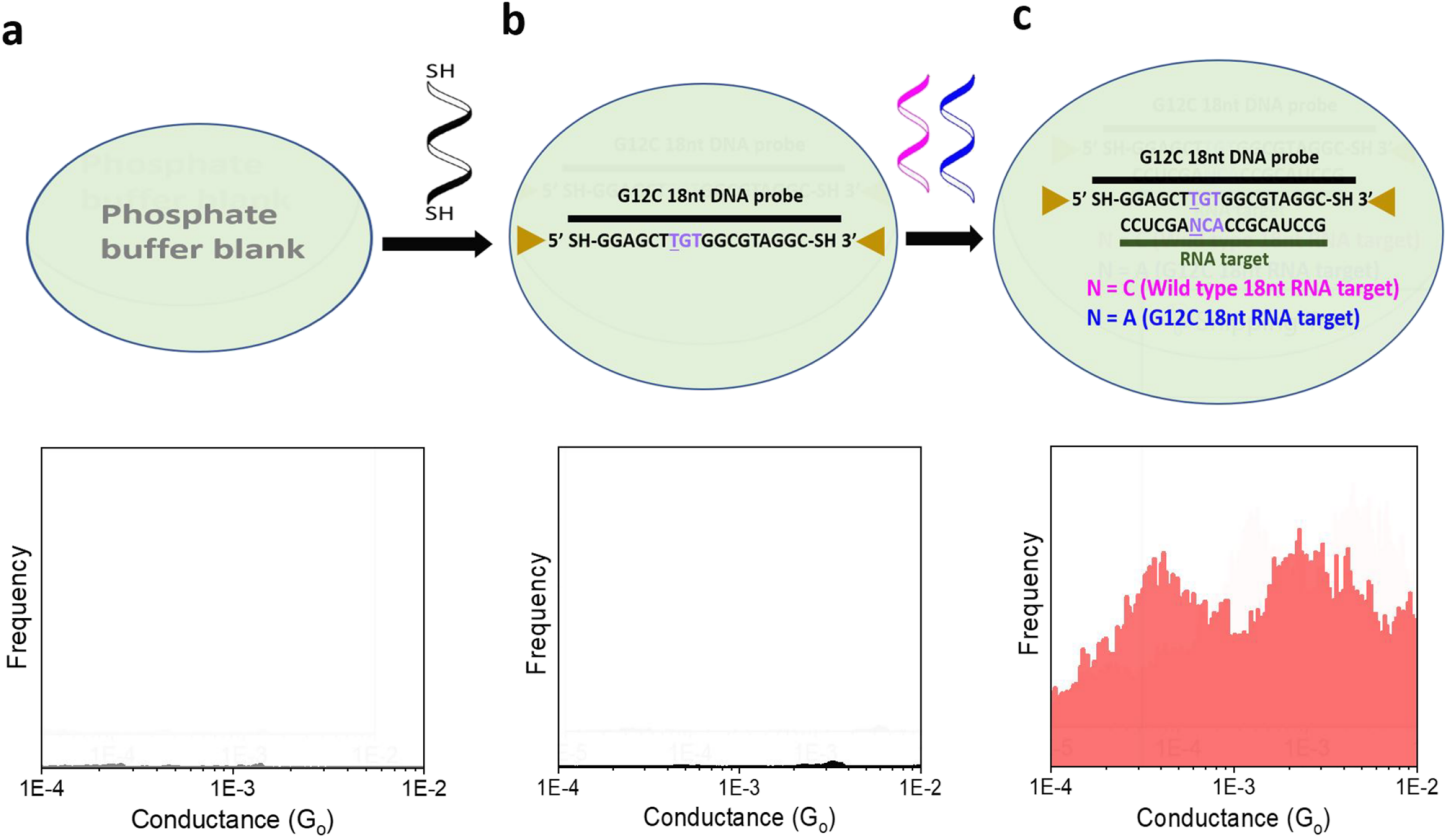
High specificity of the STMBJ approach for cancer detection: In situ hybridization for perfect match G12C. (**a**) Histogram for blank phosphate buffer (3 from a total 1150 curves acquired). (**b**) Histogram for G12C DNA probe (3 from a total 661 curves acquired). (**c**) Conductance histogram including both G12C and wild-type DNA:RNA hybrids (111 from a total 5631 curves acquired).

After demonstrating the possibility of electrical detection of individual cancer biomarkers, we turn to *in situ* hybridization experiments in the presence of mutant and wild-type RNA sequences to further challenge the specificity of this biosensing method. Figure 3 shows the results of the experiments on the G12C mutation. We define specificity as the ability to differentiate perfect match hybrids from mismatched hybrids or, more simply, the ability to discriminate the cancer biomarker from the wt KRAS sequence. We started with a buffer control experiment that did not show a conductance peak (Fig. 3a). Then we injected the thiolated DNA probe, resulting in a similar featureless conductance histogram (Fig. 3b), and finally added a mixture of mutant G12C and wt KRAS sequences. The final histogram in Fig. 3c shows two molecular conductance peaks, matching the conductance values obtained in Fig. 2c, and thus maintaining the peak separation between signals coming from cancer biomarker sequences and wt KRAS present in virtually any human-derived RNA sample. This shows that this single-molecule electrical biosensor has high specificity, enabling the detection of cancer biomarker sequences and paving the way for future applications where multiple sequences could be detected in parallel for diagnostic purposes^20^.

### Sensitivity: single-molecule detection

**Figure 4:**
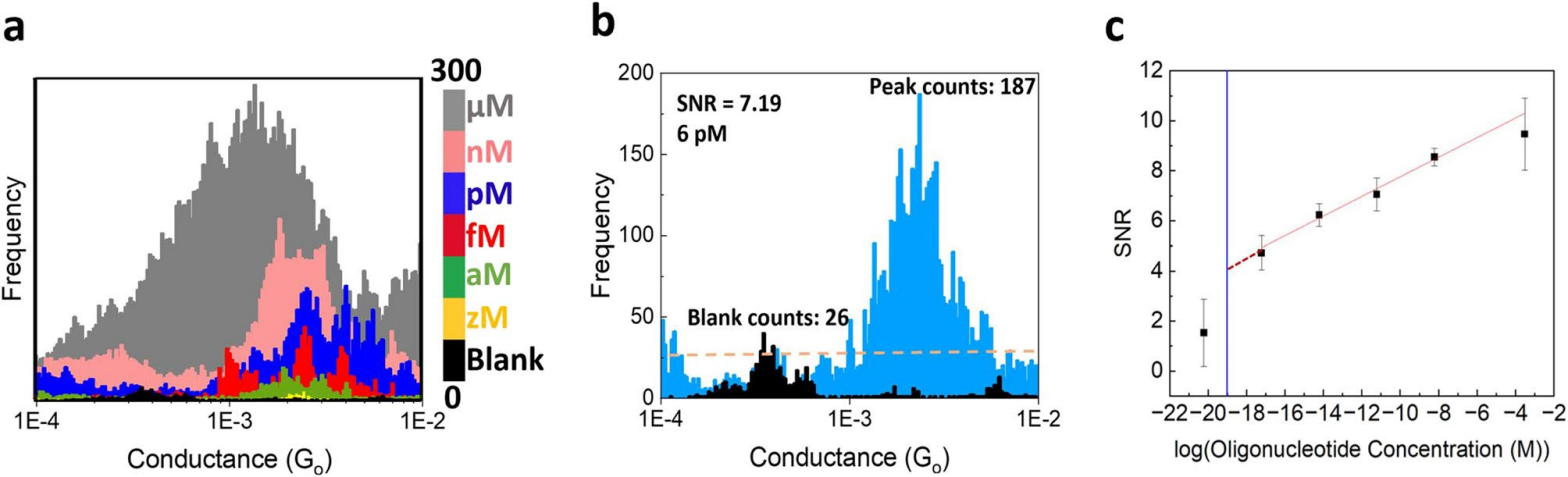
High sensitivity of the STMBJ approach for cancer detection. (**a**) Conductance histograms for G12C titration experiments (concentration varies from 300 $\upmu \hbox{M}$ to 0. The control experiment in phosphate buffer solution (black) shows no peaks in the histogram. (**b**) Limit of Detection (LoD), Example of SNR calculation for a 6 pM concentration sample. (**c**) Average SNR for each concentration (with a linear fit) to get the concentration for a $\hbox{SNR} = 3$, Blue vertical line: theoretical concentration where a single molecule is present in the sample volume: around 0.1 aM).

To evaluate the sensitivity of the approach, we performed titration experiments on KRAS G12C to determine the limit of detection (LOD) of the system. As Fig. 4a shows, we obtained conductance measurements for the 18-base pair KRAS G12C perfect match DNA:RNA hybrids at varying concentrations, ranging from 6 zM (zepto molar, $10^{-21}$ M) to 300 $\upmu \hbox{M}$. The control experiment histogram corresponding to a buffer blank is shown in black. We define the LOD as the minimum target concentration providing a signal-to-noise ratio (SNR) of at least 3. We obtained SNR by analyzing each histogram separately and calculating the ratio between the noise count (baseline count) and the peak height count at the conductance peak (see Fig. 4b for an example). Figure 4c shows the SNR obtained for the different target RNA concentrations. A vertical blue line indicates the single-molecule limit (theoretical concentration, 0.1 aM), which crosses the trend obtained in our experiments around an SNR of 4, demonstrating that the LOD is effectively an individual molecule. This LOD is the lowest obtained with this kind of biosensor, since the lowest to date was around 20 aM^11^. At lower concentrations than the LOD, the results were similar to those of control buffer experiments and the SNR value became stochastic (see the yellow zM histogram in Fig. 4a).

These titration experiments confirm the superior sensitivity and the single-molecule nature of the biosensor, paving the way for future applications in cancer screening and other diseases, after this method becomes miniaturized and automatized in the future^11,18,35^.

In summary, in this work, we present a fully electrical proof-of-concept biosensor for detecting RNA cancer biomarker sequences in solution at the single-molecule level. This study represents the first application of single-molecule measurements to a sequence from human origin. Also, it constitutes a novel exploration of electrical detection for individual cancer biomarker molecules. We demonstrate that we can distinguish KRAS RNA cancer biomarker sequences in solution from wild-type counterparts that differ in only one base. Our results indicate that the biomolecular conductance signal can be several times higher for the mutant cancer biomarkers hybridized to the DNA probe, allowing for a high signal-to-noise ratio. Moreover, this extremely specific system can simultaneously detect and differentiate multiple human RNA targets, such as healthy wild-type and mutated RNA targets. The limit of detection from titration experiments is effectively an individual molecule. The limit of detection achieved in this study establishes a new benchmark as the lowest ever reported, underscoring its exceptional sensitivity. This nanobiosensor paves the way for future inexpensive, highly sensitive, and easily operable label-free miniaturized devices for future use as a high throughput tool if key challenges in miniaturization are solved. This biosensor can potentially improve the early-stage cancer screening process through liquid biopsies.

## Methods

### Sequence and bioinformatics analysis

With the help of the literature^21,22^, we chose KRAS mutations as candidate cancer biomarkers for the study. The KRAS Exon 2 sequence was provided by UMass Medical School collaborators (Prof. M. Green group, data in supplementary Fig. S1). We selected two oligonucleotide sequences from the KRAS Exon 2 sequence as DNA probes. These two sequences contained G12C and G12V mutations, with the mutated base appearing in the center of the sequence. Both DNA probes consisted of 18 nt bases and a single-base mutation. Using the IDT Oligo Analyzer tool^31^, we obtained GC%, melting temperatures, and secondary structure information for each selected oligonucleotide sequence (Results in supplementary Table S1). The NCBI Blast tool^30^ was used to find the minimum specific lengths for DNA probes, while aligning the interesting sequences with the human genomic and transcript database (example results in supplementary Fig. S2) to check specificity.

### Sample and solution preparation

The designed DNA probes and RNA target oligonucleotide sequences (18 bases) were purchased from Biosynthesis Inc. (USA). While designing the DNA probes, thiol linkers and C6 spacers are added to the same DNA probe oligonucleotide on 5’ and 3’ ends. The oligonucleotides were received in powdered form, which was then spun down and resuspended in nuclease-free water. Next, the thermal hybridization process was carried out by heating a mixture of the DNA probe and RNA target in a phosphate buffer (100 mM final concentration) medium to 80 °C in a water bath and then allowing the mixture to cool down to room temperature for several hours. Perfectly matched DNA:RNA hybrids were formed by hybridizing mutated DNA probes with their complementary RNA targets in a phosphate buffer (100 mM final concentration) medium. Mismatched DNA:RNA hybrids were formed by hybridizing mutated DNA probes with the respective wild-type RNA targets in a phosphate buffer (100 mM final concentration) medium.

All the glassware and Teflon cells were cleaned using the piranha solution, made by adding 98% $\hbox{H}_2\hbox{SO}_4$ and 30% $\hbox{H}_2\hbox{O}_2$ in a 3:1 v/v ratio (CAUTION: Piranha solution is highly corrosive and dangerous). All other parts of the STM cell were cleaned with $(\hbox{CH}_3)_2\hbox{CO}$ (HPLC plus) and $\hbox{C}_2\hbox{H}_6\hbox{O}$ (pure). The substrate was an Au single crystal (Goodfellow), which was electropolished in 0.1 M $\hbox{H}_2\hbox{SO}_4$ using two thin loops of gold wire as electrodes before each experiment. The Au single crystal was placed on top of one gold wire loop (working electrode), and another gold wire loop (counter electrode) was placed directly above the single crystal, approximately 0.5 cm away. The working and counter electrodes were connected to the DC power supply, where about 10 V and 1 A current were applied for about 30 s, until the top layer of the Au single crystal turned an orange–brown color. Then, the oxidized Au single crystal was rinsed in Milli-Q water about five times. The Au single crystal was then soaked in 1 M HCl solution for about 3 min until the crystal returned from the orange–brown color to the shiny gold color, and again rinsed with Milli-Q water for about three times. Finally, the clean Au single crystal was air dried, before being annealed using a butane flame for about 1 min.

A 100 mM phosphate buffer was used for all experiments. $\hbox{Na}_2\hbox{HPO}_4$ and $\hbox{NaH}_2\hbox{PO}_4$ were used to prepare the 100 mM phosphate buffer (pH 7.4) with Milli-Q water. Then the buffer solution was filtered with 20 nm pore size filters (Whatman Anotop 25 plus) before use in experiments. Chemicals such as $(\hbox{CH}_3)_2\hbox{CO}$ (HPLC plus), $\hbox{C}_2\hbox{H}_6\hbox{O}$ (pure), $\hbox{H}_2\hbox{SO}_4$, HCl, $\hbox{Na}_2\hbox{HPO}_4$, and $\hbox{NaH}_2\hbox{PO}_4$ were purchased from Sigma-Aldrich.

### STM-break junction measurements

All experiments were performed using a Pico-STM Molecular Imaging head connected to a Digital Instruments Nanoscope IIIa controller and were performed at room temperature. The 10 nA/V preamplifier and a bias voltage of 100 mV and 20 mV (for some titration experiments) were used for experiments. A control phosphate buffer experiment (main text Fig. 1d black histogram) was performed before each experiment to ensure no contaminants were present in the cell. In a normal DNA:RNA hybrid experiment, a small sample volume was added to the STM cell to achieve about $\upmu \hbox{M}$ final concentrations for the conductance measurements. Thiol groups in the DNA probes were protected as disulfide bonds, and TCEP (Tris (2-carboxyethyl) phosphine) was used to reduce the disulfide bond to produce free thiol groups that can be attached to the Au surface and the Au tip. TCEP completely and selectively reduces disulfides over a wide pH range, requiring less than 5 min at room temperature.

The STM tip was made by cutting 0.25 mm diameter gold wire (Goodfellow) at about a 45° angle and then coating it with Apiezon wax (Apiezon products). Waxing the tip helps to minimize the exposed surface area of the tip, reducing leakage of faradaic currents.

With the STM-BJ method^10^, the STM tip was repeatedly brought into and out of contact with the Au substrate and then pulled away, forming a tip-substrate gap that can be bridged by a molecule at a nanometric length. A customized LabVIEW program^36^ was used to control the movement of the STM tip and acquire data. Approximately four thousand current-distance traces were recorded during a single experiment. We observed steps in some traces when a molecule was bound between the Au tip and the Au substrate.

### Statistical analysis

A LabVIEW program was used to analyze the thousands of collected traces^36^. This program performs a logarithmic binning of each recorded current-distance trace. The algorithm goes through all the data curves and selects curves that meet specific criteria. In the LabVIEW program, we define these parameters as a step height of 1, a decay value of 0.02, and a length comprising 99% of the curve. For example, when treating one of the KRAS G12V mismatch datasets, we initially acquired 5024 curves from the STM-BJ experiments. Employing the parameters mentioned above during the initial automated process in LabVIEW, the program identified and selected 3354 curves. This was further refined with a manual selection, discarding noisy curves and accepting only curves with clear plateaus. This process selected 75 curves from the total 5024 traces, exhibiting a selectivity of 1.5%. Following the manual selection process, all the selected curves with steps were added together to obtain a semi-logarithmic conductance histogram representing the most probable conductance of the specific molecular junction.

## Supplementary Information

Supplementary Information.

## Data Availability

All data are available in the main text, the supplementary materials, or provided on demand.
