# Supplementary material for: Electrical detection of RNA cancer biomarkers at the single-molecule level
Source: Sci Rep. 2023 Aug 1;13:12428. doi: 10.1038/s41598-023-39450-6 (PMC10393997; doi:10.1038/s41598-023-39450-6)
Supplement: Supplementary file 1 — Supplementary Information. [file 41598_2023_39450_MOESM1_ESM.pdf]

Supplementary Information for

Electrical detection of RNA cancer biomarkers at the single-molecule level

Keshani G. Gunasinghe Pattiya Arachchillage, Subrata Chandra, Ajoke Williams, Patrick Piscitelli, Jennifer Pham, Aderlyn Castillo, Lily Florence, Srijith Rangan, Juan M. Artes Vivancos*

Department of Chemistry, University of Massachusetts Lowell, Lowell, MA, 01854, USA

Correspondence to: *juan_artesvivancos@uml.edu

This PDF file includes:

Figs. S1 to S3
Table S1

a

KRAS Exon 2 sequence

GCCTGCTGAAA***<u>ATG</u>***ACTGAATATAAACTTGTGGTAGTTGGAGCT***<u>GGT</u>***GGCGTAGGCA
AGAGTGCCTTGACGATACAGCTAATTCAGAATCATTTTGTGGACGAATATGATCCAAC
AATAGAG

The sequence in black is Exon 2 of wild-type KRAS. The bold, italic, and underlined bases are the start codon (ATG) and G12 codon (GGT) of wild-type KRAS. KRAS mutations occur at the G12 codon (e.g., G12C, G12V, etc.)

b

Selected Oligonucleotide sequences for DNA probes:

For G12V mutation: GGAGCTG**<u>T</u>**TGGCGTAGGC (18 nt)
For G12C mutation: GGAGCT**<u>T</u>**GTGGCGTAGGC (18 nt)

The base underlined in each oligonucleotide sequence is the place where the mutation occurs, and each mutation presents in the middle of the oligonucleotide sequence

Fig. S1. Selecting appropriate sequences for the study. a, KRAS Exon 2 sequence **b,** selected oligonucleotide sequences as DNA probes for the study

a

RID: N0AZF94M013
Job Title: G12C 18nt sequence (including all genomes)
Program: BLASTN
Database: Human G+T (2 databases)
Query #1: Query ID: lcl|Query_46647 Length: 18

Sequences producing significant alignments:

Description	Scientific Name	Common Name	Taxid	Query cover	Per. Ident	Accession
PREDICTED: Homo sapiens uncharacterized LOC124904956...	Homo sapiens	human	9606	77%	100.00	XR_007067720.1
PREDICTED: Homo sapiens KRAS proto-oncogene, GTPase (KRAS),...	Homo sapiens	human	9606	100%	94.44	XM_047428826.1
**Homo sapiens KRAS proto-oncogene, GTPase (KRAS), transcript...**	**Homo sapiens**	**human**	**9606**	**100%**	**94.44**	**NM_001369786.1**
**Homo sapiens KRAS proto-oncogene, GTPase (KRAS), transcript...**	**Homo sapiens**	**human**	**9606**	**100%**	**94.44**	**NM_001369787.1**
**Homo sapiens KRAS proto-oncogene, GTPase (KRAS), transcript...**	**Homo sapiens**	**human**	**9606**	**100%**	**94.44**	**NM_033360.4**
**Homo sapiens KRAS proto-oncogene, GTPase (KRAS), transcript...**	**Homo sapiens**	**human**	**9606**	**100%**	**94.44**	**NM_004985.5**
Homo sapiens MAP kinase activating death domain (MADD),...	Homo sapiens	human	9606	72%	100.00	NR_164835.1
Homo sapiens MAP kinase	Homo sapiens	human	9606	72%	100.00	NR_164836.1

activating death domain (MADD),...						
Homo sapiens MAP kinase activating death domain (MADD),...	Homo sapiens	human	9606	72%	100.00	NR_164837.1
Homo sapiens MAP kinase activating death domain (MADD),...	Homo sapiens	human	9606	72%	100.00	NR_164839.1

RID: N0AZF94M013
Job Title: G12C 18nt sequence (including all genomes)
Program: BLASTN
Database: Human G+T (2 databases)
Query #1: Query ID: lcl|Query_46647 Length: 18

Sequences producing significant alignments:

Description	Scientific Name	Common Name	Taxid	Max Score	Total Score	Query cover	E Value	Per. Ident	Acc. Len	Accession
PREDICTED: Homo sapiens uncharacterized LOC124904956...	Homo sapiens	human	9606	28.2	28.2	77%	25	100.00	1490	XR_007067720.1
PREDICTED: Homo sapiens KRAS proto-oncogene, GTPase (KRAS),...	Homo sapiens	human	9606	28.2	28.2	100%	25	94.44	5410	XM_047428826.1
Homo sapiens KRAS proto-oncogene, GTPase (KRAS), transcript...	Homo sapiens	human	9606	28.2	28.2	100%	25	94.44	5417	NM_001369786.1
Homo sapiens KRAS proto-oncogene, GTPase (KRAS), transcript...	Homo sapiens	human	9606	28.2	28.2	100%	25	94.44	5293	NM_001369787.1
Homo sapiens KRAS proto-oncogene, GTPase (KRAS), transcript...	Homo sapiens	human	9606	28.2	28.2	100%	25	94.44	5430	NM_033360.4
Homo sapiens KRAS proto-oncogene, GTPase (KRAS), transcript...	Homo sapiens	human	9606	28.2	28.2	100%	25	94.44	5306	NM_004985.5
Homo sapiens MAP kinase activating death domain (MADD),...	Homo sapiens	human	9606	26.3	26.3	72%	101	100.00	6087	NR_164835.1
Homo sapiens MAP kinase activating death domain (MADD),...	Homo sapiens	human	9606	26.3	26.3	72%	101	100.00	5954	NR_164836.1
Homo sapiens MAP kinase activating death domain (MADD),...	Homo sapiens	human	9606	26.3	26.3	72%	101	100.00	5276	NR_164837.1
Homo sapiens MAP kinase activating death domain (MADD),...	Homo sapiens	human	9606	26.3	26.3	72%	101	100.00	5852	NR_164839.1
Homo sapiens MAP kinase activating death domain (MADD),...	Homo sapiens	human	9606	26.3	26.3	72%	101	100.00	6045	NR_164840.1
Homo sapiens MAP kinase activating death domain (MADD),...	Homo sapiens	human	9606	26.3	26.3	72%	101	100.00	6278	NR_164841.1
Homo sapiens MAP kinase activating death domain (MADD),...	Homo sapiens	human	9606	26.3	26.3	72%	101	100.00	6321	NR_164842.1
Homo sapiens MAP kinase activating death domain (MADD),...	Homo sapiens	human	9606	26.3	26.3	72%	101	100.00	5458	NR_164838.1
Homo sapiens MAP kinase activating death domain (MADD),...	Homo sapiens	human	9606	26.3	26.3	72%	101	100.00	5696	NM_001376651.1

b

RID: SWCJD4VM013
Job Title: G12C 17nt sequence (including all genomes)
Program: BLASTN
Query: None ID: lcl|Query_52815(nucleic acid) Length: 17
Database: Human G+T (2 databases)

Sequences producing significant alignments:

Description	Scientific Name	Common Name	Taxid	Query cover	Per. Ident	Accession
PREDICTED: Homo sapiens uncharacterized LOC124904956...	Homo sapiens	human	9606	82%	100.00	XR_007067720.1
PREDICTED: Homo sapiens KRAS proto-oncogene, GTPase (KRAS),...	Homo sapiens	human	9606	100%	94.12	XM_047428826.1
Homo sapiens MAP kinase activating death domain (MADD),...	Homo sapiens	human	9606	76%	100.00	NR_164835.1
Homo sapiens MAP kinase activating death domain (MADD),...	Homo sapiens	human	9606	76%	100.00	NR_164836.1
Homo sapiens MAP kinase activating death domain (MADD),...	Homo sapiens	human	9606	76%	100.00	NR_164837.1
Homo sapiens MAP kinase activating death domain (MADD),...	Homo sapiens	human	9606	76%	100.00	NR_164839.1
Homo sapiens MAP kinase activating death domain (MADD),...	Homo sapiens	human	9606	76%	100.00	NR_164840.1
Homo sapiens MAP kinase activating death domain (MADD),...	Homo sapiens	human	9606	76%	100.00	NR_164841.1

Homo sapiens MAP kinase activating death domain (MADD),...	Homo sapiens	human	9606	76%	100.00	NR_164842.1
Homo sapiens MAP kinase activating death domain (MADD),...	Homo sapiens	human	9606	76%	100.00	NR_164838.1

RID: SWCJD4VM013
Job Title:G12C 17nt sequence (including all genomes)
Program: BLASTN
Query: None ID: lcl|Query_52815(nucleic acid) Length: 17
Database: Human G+T (2 databases)

Sequences producing significant alignments:

Description	Scientific Name	Common Name	Taxid	Max Score	Total Score	Query cover	E Value	Per. Ident	Acc. Len	Accession
PREDICTED: Homo sapiens uncharacterized LOC124904956...	Homo sapiens	human	9606	28.2	28.2	82%	25	100.00	1490	XR_007067720.1
PREDICTED: Homo sapiens KRAS proto-oncogene, GTPase (KRAS),...	Homo sapiens	human	9606	26.3	26.3	100%	101	94.12	5410	XM_047428826.1
Homo sapiens MAP kinase activating death domain (MADD),...	Homo sapiens	human	9606	26.3	26.3	76%	101	100.00	6087	NR_164835.1
Homo sapiens MAP kinase activating death domain (MADD),...	Homo sapiens	human	9606	26.3	26.3	76%	101	100.00	5954	NR_164836.1
Homo sapiens MAP kinase activating death domain (MADD),...	Homo sapiens	human	9606	26.3	26.3	76%	101	100.00	5276	NR_164837.1
Homo sapiens MAP kinase activating death domain (MADD),...	Homo sapiens	human	9606	26.3	26.3	76%	101	100.00	5852	NR_164839.1
Homo sapiens MAP kinase activating death domain (MADD),...	Homo sapiens	human	9606	26.3	26.3	76%	101	100.00	6045	NR_164840.1
Homo sapiens MAP kinase activating death domain (MADD),...	Homo sapiens	human	9606	26.3	26.3	76%	101	100.00	6278	NR_164841.1
Homo sapiens MAP kinase activating death domain (MADD),...	Homo sapiens	human	9606	26.3	26.3	76%	101	100.00	6321	NR_164842.1
Homo sapiens MAP kinase activating death domain (MADD),...	Homo sapiens	human	9606	26.3	26.3	76%	101	100.00	5458	NR_164838.1
Homo sapiens MAP kinase activating death domain (MADD),...	Homo sapiens	human	9606	26.3	26.3	76%	101	100.00	5696	NM_001376651.1
Homo sapiens MAP kinase activating death domain (MADD),...	Homo sapiens	human	9606	26.3	26.3	76%	101	100.00	5937	NM_001376601.1
Homo sapiens MAP kinase activating death domain (MADD),...	Homo sapiens	human	9606	26.3	26.3	76%	101	100.00	5937	NM_001376578.1
Homo sapiens MAP kinase activating death domain (MADD),...	Homo sapiens	human	9606	26.3	26.3	76%	101	100.00	5812	NM_001376594.1
Homo sapiens MAP kinase activating death domain (MADD),...	Homo sapiens	human	9606	26.3	26.3	76%	101	100.00	5781	NM_001376604.1

Fig. S2. NCBI BLASTN[1] results demonstrating that the chosen G12C 18nt sequence is specific for the KRAS proto-oncogene and G12C 17nt is nonspecific. a, G12C 18nt sequence (GGAGCTTGTGGCGTAGGC); Region in the black and bold shows the chosen sequence is specific for KRAS proto-oncogene (Table continues...) **b,** G12C 17nt sequence (GGAGCTTGTGGCGTAGG); nonspecific for KRAS proto-oncogene (Table continues...) (Both **a** and **b** represent a table with all important information and a snapshot from NCBI Blast work)

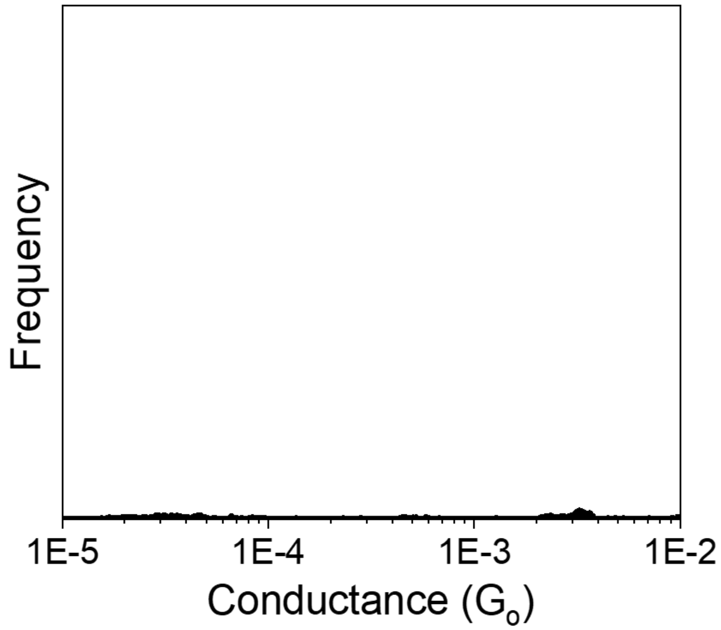

Fig. S3. Conductance histogram for a control experiment. Experiment for G12C 18nt DNA probe (GGAGCTTGTGGCGTAGGC)

a

**Sequence**	**G12V 18nt**<br>GGAGCTG<u>**T**</u>TGGCGTAGGC	**G12C 18nt**<br>GGAGCT<u>**T**</u>GTGGCGTAGGC
**GC%**	66.7	66.7
**Tm (^{o}C)**	59	59

b

**Sequence**	**Secondary structures and their Tm**	
**G12V 18nt**<br>GGAGCTG<u>**T**</u>TGGCGTAGGC	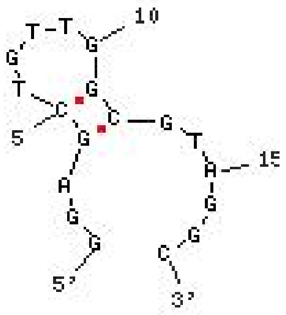<br>**35.5 ^{o}C**	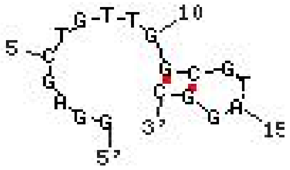<br>**31.3 ^{o}C**
**G12C 18nt**<br>GGAGCT<u>**T**</u>GTGGCGTAGGC	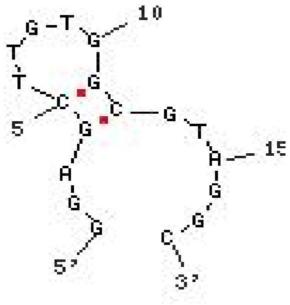<br>**35.5 ^{o}C**	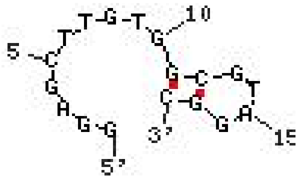<br>**31.3 ^{o}C**

Table S1. Sequence analysis using IDT OligoAnalyzer tool. a, IDT OligoAnalyzer[2] results (GC% and Tm) for selected G12C and G12V oligonucleotide sequences **b,** Secondary structures and their melting temperatures for selected G12C and G12V oligonucleotide sequences (using the default parameters in the IDT OligoAnalyzer tool[2])

References for Supplementary Information

1 NCBI blast tool. *NCBI blast tool*, <https://blast.ncbi.nlm.nih.gov/Blast.cgi?PROGRAM=blastn&PAGE_TYPE=BlastSearch&LINK_LOC= blasthome> (2022).

2 Tool, I. O. *IDT OligoAnalyzer Tool*, <https://www.idtdna.com/calc/analyzer> (2022).
